# Improving Equity in Deep Learning Medical Applications with the Gerchberg-Saxton Algorithm

**DOI:** 10.1007/s41666-024-00163-8

**Published:** 2024-02-28

**Authors:** Seha Ay, Michael Cardei, Anne-Marie Meyer, Wei Zhang, Umit Topaloglu

**Affiliations:** 1 Department of Biomedical Engineering, Wake Forest School of Medicine, Winston-Salem, NC USA; 2 Department of Cancer Biology, Wake Forest School of Medicine, Winston-Salem, NC USA; 3 Department of Epidemiology, University of North Carolina, Chapel Hill, NC USA; 4 National Cancer Institute, Shady Grove, Rockville, MD USA

**Keywords:** Deep learning, Medical decision-making, Racial bias mitigation, MIMIC-III, Mortality rate prediction

## Abstract

Deep learning (DL) has gained prominence in healthcare for its ability to facilitate early diagnosis, treatment identification with associated prognosis, and varying patient outcome predictions. However, because of highly variable medical practices and unsystematic data collection approaches, DL can unfortunately exacerbate biases and distort estimates. For example, the presence of sampling bias poses a significant challenge to the efficacy and generalizability of any statistical model. Even with DL approaches, selection bias can lead to inconsistent, suboptimal, or inaccurate model results, especially for underrepresented populations. Therefore, without addressing bias, wider implementation of DL approaches can potentially cause unintended harm. In this paper, we studied a novel method for bias reduction that leverages the frequency domain transformation via the Gerchberg-Saxton and corresponding impact on the outcome from a racio-ethnic bias perspective.

## Introduction

Machine learning’s ubiquitous presence in a variety of domains has had a profound impact on many industries leading to advancements and previously unfeasible progress. Specifically, in healthcare, deep learning has emerged as a promising tool in drug discovery [1], early diagnosis [2], advancements in medical imaging [3], and personalized treatments [4]. The growing prominence and initial results of deep learning have unearthed significant drawbacks and limitations from interpretability to data availability, and more importantly inherent biases. Recently, the artificial intelligence research community has realized the undesired impact and potential danger of bias and fairness in healthcare. While complete elimination may not be yet possible, mitigation and counter measures may enable DL adaptation in clinical applications in a relatively considerate manner.

In the context of machine learning, bias refers to the presence of systematic inaccuracies or distortions that result in a disadvantage to a certain group of people. In contrast, fairness is the absence of prejudice based on an inherited or acquired characteristic [5]. Bias in machine learning can be represented in various ways, including data-to-algorithm bias, which arises from a biased dataset resulting in a subsequent bias in algorithmic outcomes; algorithm-to-user bias, which occurs when the machine learning algorithm itself is biased; and user-to-data, which the users’ inherited bias is reflected in the data generated. In this study, the data-to-algorithm bias is investigated and mitigated. Sampling bias occurs when the samples used for model training are not representative of the source population. Overall, caution is always needed as the trends and patterns estimated for one population do not necessarily generalize to other external populations. This is especially frequent in healthcare where data is typically skewed toward a specific characteristic of the population serviced by a healthcare facility and its providers in a geographic location with corresponding risk or socioeconomic factors. Particularly, the representation bias arises when the data does not accurately represent the true (or uniform) distribution of the population, and similarly, population bias occurs when the distribution of the training data does not represent the population the model was intended for [5]. Ensuring fairness is crucial for the deployment of a safe (i.e., remediation of unintended harm) and ethical machine learning application since failure may cause potentially dangerous and life-altering events. Especially in medicine, misdiagnosis and incorrect treatment recommendation due to unmitigated bias could lead to unintended and severe consequences for patients.

In this paper, we propose a method for racial bias mitigation in DL classification based on advanced frequency domain operations by utilizing the Gerchberg-Saxton (GS) algorithm. This method operates by distributing the information carried among features utilized in model training more uniformly that is achieved by leveraging the frequency domain magnitude equalization feature of the GS algorithm. Specifically, we demonstrate that the racial bias caused by the information embedded in the data features can be minimized and results in more consistent models with more uniform accuracy per race category. This approach has the potential to offer an innovative solution for resolving discrepancies in model results that arise from the uneven representation of population. Hence, improving model performance for underrepresented populations has the potential to improve equity in healthcare.

## Related Work

As the utilization of Artificial Intelligence (AI) has rapidly exploded in recent years, the fairness and bias resulting from these approaches are crucial considering the importance of equitable decision-making processes and potential harm. Various methodologies and frameworks have been proposed to overcome such challenges, each with unique advantages with corresponding challenges.

A widely discussed method to overcome these problems, data augmentation focuses on generating synthetic samples to reinforce the representation of under-represented groups in the training dataset. While this approach has proven some success, the data augmentation method often carries concerns on generating an accurate representation of samples for specific populations, consequentially making it less effective for comprehensive bias mitigation [6]. Some other works have further delved into the potential pitfalls of this approach, emphasizing the need for careful implementation to avoid inadvertently introducing new biases [7, 8].

Another noteworthy approach is adversarial learning, where a predictor and adversary models are trained simultaneously. In this approach, the objective is to sharpen the predictor’s ability to yield the desired outcome while mitigating the adversary’s efficiency in predicting the target variable. This strategy has shown effectiveness in bias mitigation, especially when compared with various fairness measurements. Moreover, to address its inherent limitations in assessing fairness across all testing cohorts, a novel fairness prediction scheme is introduced, allowing users to evaluate the model’s alignment with their fairness criteria [9]. However, despite its potential, the demand for the computational power and the method’s effectiveness on complex datasets warrant further exploration [10].

On the topic of fairness constraints, there’s ongoing research concentrating on their integration within learning processes to guarantee just outcomes. However, there is a lot of debate about which constraints are the most appropriate with no absolute conclusions, making this approach an upcoming area of discussion and innovation [5].

In the realm of deep reinforcement learning, Yang et al. demonstrated a novel approach by focusing on algorithmic fairness to achieve equal odds. Although their results showed a noteworthy decline in bias, the model failed to fulfill the equalized odd requirements [11]. Furthermore, its model-specific nature makes it less generalizable for wider applications, especially in biomedicine. However, this method’s merits cannot be overlooked, as it offers fresh insights into the broader dialogue on fairness in AI.

Our contribution to this ongoing discourse is a model-agnostic method that targets bias directly within the data. At the heart of our approach is the Gerchberg-Saxton (GS) algorithm, traditionally utilized in computer vision for optic and signal processing applications. The GS algorithm’s prowess lies in its ability to optimize information distribution, making it an invaluable tool in our quest for fairness. In the context of AI fairness, our innovative application of the GS algorithm promises a new pathway to tackle bias, drawing from its established efficacy in other domains.

In essence, the landscape of bias mitigation in AI is diverse and dynamic. While significant strides have been made, the quest for the most effective and universally applicable methodology continues. Our work, building upon the foundation laid by previous researchers, introduces a promising avenue, leveraging the proven capabilities of the GS algorithm in a novel context.

## Methodology

### Clinical Data Source and Study Population

This study utilized the Medical Information Mart for Intensive Care (MIMIC) III version 1.4 database, which has been widely used in research for developing and testing machine learning algorithms and predictive models for various clinical applications, such as mortality prediction, sepsis detection, and disease phenotyping [12]. The database is created and maintained by the MIT Laboratory for Computational Physiology. The data contains de-identified health data from electronic medical records, laboratory information systems, and bedside monitors from over 40,000 patients who were admitted to intensive care units (ICUs) at the Beth Israel Deaconess Medical Center in Boston, Massachusetts, USA between 2001 and 2012 [12].

In this study, we employed the MIMIC-III dataset to predict mortality rates while demonstrating bias across different racio-ethnic groups. Thirty vital sign features were leveraged to train a DL classification model that predicts patient mortality rates within 24 h of admission. All feature values were selected from score equivalents of the actual feature values ranging from 0 to 22 or features indicating probability scores to minimize numeric variance. Patients with any missing values were removed from the dataset, and the final sub-selected dataset consists of 13,980 patients from 36 different racial and ethnic groups. To produce a dataset more conducive to analysis and observation, the 36 different ethnic groups were re-classified based on patients’ self-reported common ancestral heritage [13, 14]. The resulting dataset consisted of 9814 patients categorized as “European American” (EA), 1690 patients categorized as “African American” (AA), 346 patients categorized as “Eastern Asian American” (EEA), 641 patients categorized as “Hispanic American”, and 1489 patients who identified as “Unkown” or declined to report were categorized as “Others” (OTH). The MIMIC-III data has previously been shown to have a potential bias that can adversely impact the accuracy of predictive models [13, 14]. Therefore, our study demonstrates that we can mitigate racio-ethnic bias allowing for more equitable and unbiased estimates. It is crucial to note that the categorization was based solely on patients’ self-reported ethnicity and was used for research purposes only.

To evaluate the efficacy of the Gerchberg-Saxton (GS) algorithm in bias mitigation on the MIMIC-III, we conducted an analysis of the models’ accuracy, demographic parity, and error rate parity fairness constraints among the sensitive population groups using true positive, true negative, false positive and false negative rates obtained individually from both the benchmark dataset, and the GS applied dataset. The model was trained to classify patients who passed away within 24 h of admission as “True” (1) and patients who passed away longer than 24 h after admission or an unspecified time as death as “False” (0). These experiments were repeated with three different population cohorts, which are constructed using the same patients in different sampling settings to produce consistent and reproducible results. Tests were run five times for each cohort resulting in a total of fifteen repetitions.

### Gerchberg-Saxton Algorithm

The Gerchberg-Saxton (GS) algorithm is an iterative phase retrieval technique developed for determining the phase of a light or electron beam from its intensity distributions in two transverse planes [15]. It is frequently used in holographic imaging to extract the phase patterns of images from corresponding intensity patterns, making it a valuable tool in image processing for reconstructing images with unknown phase patterns [16]. The algorithm takes the magnitudes of the sample image ($\chi$) and the corresponding Fourier transform of the diffraction intensities ($X$) as input parameters to estimate the phase pattern of the input image. The GS algorithm iteratively cycles between the image and diffraction planes using Fourier and Inverse Fourier transforms until an estimation for the phase pattern of the input image is obtained. We use the standard definition, in which the Discrete Fourier Transform (DFT) of $f$ is $F$ and the Inverse DFT (IDFT) of $F$ is $f$ where

1 $$F\left[m\right]={\sum }_{n=0}^{N-1}f\left[n\right]{e}^{-i2\pi mn/N}$$

2 $$f\left[n\right]=\frac{1}{N}{\sum }_{m=0}^{N-1}F{e}^{i2\pi mn/N}$$

In the mathematical representation of the GS procedures, $x$ and $X$ represent the intensity arrays for the image and diffraction, respectively, and the superscripts of $H$ and $T$ indicate the hologram and target planes, respectively. The subscript $k$ denotes the number of iterations in the GS algorithm.

3 $${\varphi }_{0}^{H}=\left[\begin{array}{ccc}{\theta }_{11}& \cdots & {\theta }_{1j}\\ \vdots & \ddots & \vdots \\ {\theta }_{i1}& \cdots & {\theta }_{ij}\end{array}\right]$$

4 $${x}_{k}^{H}=\left|f\right|{e}^{i{\varphi }_{k-1}^{H}}$$

5 $${X}_{k}^{T}=DFT\left({x}_{k}\right)=\left|{X}_{k}\right|{e}^{i{\varphi }_{k}^{H}}$$

6 $${x}_{k}^{T}=\left|F\right|{e}^{i{\varphi }_{k-1}^{T}}$$

7 $${X}_{k}^{H}=IDFT\left({x}_{k}^{T}\right)=\left|f\right|{e}^{i{\varphi }_{k}^{H}}$$

The GS algorithm begins by generating random phases between 0 and 2 $\pi$, which are then assigned to the image amplitudes using (3) and (4). Subsequently, the algorithm uses (5) to compute the DFT of the image plane and updates the phase distribution using (6). The algorithm then calculates the IDFT (7) of the diffraction plane to prepare a new set of phase distributions for the next iteration. Repeating this process for all iterations of the GS algorithm results in an improved phase distribution on each cycle, eventually leading to the best estimation on the final iteration [15–17]. The optimal phase distribution is produced within 20 cycles of the GS transformation; however, the number of cycles required may vary depending on the input matrix [18–20].

### Gerchberg-Saxton Algorithm Effect on MIMIC-III

To achieve a fairer model prediction rate, the Gerchberg-Saxton algorithm was applied to the modified MIMIC-III cohorts *before* splitting into training, test, and validation datasets. This is done to leverage GS’s information distribution property on the vital sign features utilized across the entire modified MIMIC-III cohorts and ensure equal information distribution among all the subsequent data subsets. The GS algorithm was applied for 50 cycles, transforming each element of the matrix input to its frequency domain equivalent with a magnitude and phase component. The magnitude component represents the element’s intensity, in this case, the feature value’s information, while the phase component indicates its location. During each cycle in the GS algorithm, the phase components were preserved, and the magnitude components were equalized to 1, resulting in an output matrix with a uniform distribution of feature information across the entire dataset. Moreover, preserving the phase (location) information during the GS algorithm among features increased the variance of constant-valued features based on the patient (row) they belonged in the tabular data, thus, increased the model contribution of those features on post-GS transformations. The population sample and model architecture were kept consistent for each cohort for benchmark and GS model testing.

To ensure the output matrix’s suitability for model training, we compared the input and output matrices first overall side by side, and then individually feature by feature using heatmaps (see Appendix). We evaluated the output matrix’s effect based on the preservation of the local minima and maxima locations before and after the GS algorithm to assess the transformed dataset’s ability to preserve essential features for predicting mortality rates. The model used was a Deep Neural Network (DNN) via TensorFlow 2.4.0 with ReLu activation functions and Adam optimizer. Our model adopts a sequential architecture comprising four layers. The input layer accepts data with a specified shape of (35,). Subsequently, three fully connected (dense) layers follow, where the number of nodes exhibits a reversed pyramid structure: 64 nodes in the first layer, 32 nodes in the second layer, and 16 nodes in the third layer. The final layer serves as the output layer, consisting of two units and employing the SoftMax activation function. To avoid unbalanced model training on mortality rate predictions as well as to avoid model overfitting, our DNN model is supported with balanced class weights and early stopping conditions. While the patients’ ethnic backgrounds were recorded and utilized for analysis and evaluation purposes, they were not included in model training. To minimize the computational time required by the algorithm, we have incorporated the CUDA’s *CuPy* library as an alternative to *NumPy*. This library enables GPU utilization during the Gerchberg-Saxton (GS) transformations, thereby enhancing efficiency.

### Feature Contribution on Model Training

To investigate the effect of Gerchberg-Saxton on feature contributions during model training, we used the Shapley Additive Explanations (SHAP) to provide a more definitive and substantiated view on the effectiveness of the GS algorithm in mitigating racial bias. SHAP is a mathematical concept that assigns importance values to each feature in a machine learning model to estimate its impact on the model’s output [21]. It is commonly used in machine learning to explain the output of a model by identifying the contribution of each feature to the predicted outcome [21].

The SHAP calculations were performed for all three cohorts used for data splitting to ensure consistency of the obtained results. We performed the SHAP calculations on both trained models to compare feature contributions on the model training before and after GS transformation was applied. The goal is to illustrate three main outcomes on post-GS-trained model results.

First, we expect to see a more equalized feature contribution on the model trained with the post-GS transformed dataset. This expectation can be demonstrated by a significant improvement in the effectiveness of GS transformations for bias mitigation resulting from a more uniform feature contribution on the model training after the GS transformations. Second, we expect to see a different order of feature importance on model contribution after GS transformations. Specifically, we expect to demonstrate the impact of the GS algorithm in redistributing the feature importance across different racio-ethnic groups by minimizing the ethnic discrimination effect of each feature. Lastly, we expect to observe all features used in the model training have a positive or non-zero contribution on the model training for post-GS transformations. This expected outcome illustrates that the GS applied dataset utilizes all features in model training, thus, providing a trained model more representative of the entire population.

### Information Entropy

Another measure of the effectiveness of the Gerchberg-Saxton algorithm in racio-ethnic bias mitigation is calculated via the information entropy across all trainable features using Shannon’s Entropy Theorem. Shannon entropy is a measure of the amount of variety or uncertainty in a dataset. It is commonly used in machine learning to identify informative features with higher discriminatory power. Features with higher entropy contain more information and are more likely to be relied on accurate predictions. On the other hand, features with lower entropy may be redundant or irrelevant to the task at hand [22].

Shannon entropy values of the training dataset were calculated before and after the GS transformations to measure whether the information among the features was distributed more uniformly. Considering feature values are ranging between 0 and 22, we have used logarithmic base 10 during the Shannon Entropy calculations. Although the Shannon Entropy metric cannot specifically identify the information causing bias among the racio-ethnic groups, we aimed to show a more uniform distribution of information entropy in the post-GS transformed dataset, which may indicate bias mitigation.

### Fairness Constraints

Demographic Parity and Error Rate Parity are fairness constraints or metrics often employed for the machine learning implementations. They are used to evaluate the fairness of a model or algorithm by assessing the bias in its outcomes [23]. Demographic Parity, also known as statistical or group fairness, requires that the decision or classification outcome be independent of the protected attribute, typically demographic features such as age, gender, or race. This constraint is applied to ensure that the proportion of positive outcomes is the same across all groups. Mathematically, this is expressed as P($\dot{Y}$= 1 | A = a) = P($\dot{Y}$= 1) for all a ∈ A, where $\dot{Y}$ is the predicted outcome, and A is the protected attribute [24]. The Demographic Parity Difference can be computed as the absolute difference between these probabilities to quantify the bias. The closer the value is to zero, the less the bias.

Error Rate Parity, on the other hand, requires that the error rates of the prediction should be similar across all demographic groups. It specifically considers two types of errors: false positive rate (FPR) and false negative rate (FNR). FPR Parity is achieved when P($\dot{Y}$ ≠ Y | A = a, Y = 0) = P($\dot{Y}$ ≠ Y | Y = 0) for all a ∈ A, and FNR Parity when P($\dot{Y}$ ≠ Y | A = a, Y = 1) = P($\dot{Y}$≠ Y | Y = 1) for all a ∈ A, where Y is the actual outcome [25]. The disparity in these rates between the groups is measured using the Absolute Error Rate Difference for each type of error. The calculation of these metrics is central to understanding the biases embedded in machine learning models and contributes toward building fairer and more equitable algorithms.

Our fairness evaluation centered on sensitive racio-ethnic population groups, including European-American (EA), African American (AA), East-Asian-American (EAA), Hispanic-American (HA), and Others (OTH). The fairness of the Gerchberg-Saxton (GS) algorithm in treating these sensitive groups was examined by training and testing two identical models. The benchmark model was trained on the original MIMIC-III dataset, whereas the second model was trained on a GS-transformed version of the MIMIC-III dataset. Both models were evaluated based on Demographic Parity and Error Rate Parity, key fairness metrics used to assess the balance of positive predictions and error rates across the different race-ethnic groups [24].

## Results

**Fig. 1:**
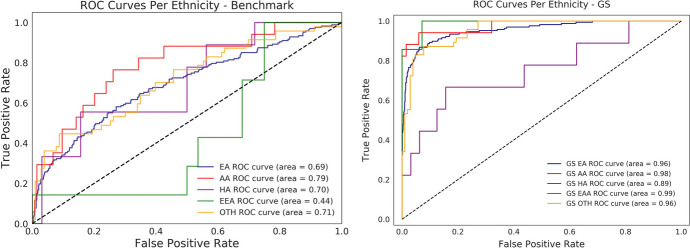
Comparative Receiver Operating Characteristic (ROC) curves per racio-ethnic groups in Benchmark and GS models. Despite the acceptable results illustrated by the benchmark model, the model trained with the GS transformed data consistently exhibits higher AUC values and more uniform prediction scores across all racio-ethnic groups, indicating enhanced predictive accuracy and less disparate model performances relative to the Benchmark model

All methods within this study have been repeated for three different population cohorts resulting in similar behavior and effects of Gerchberg-Saxton throughout. Thus, the cohort with the most concise and clear results will be shown for simplicity (Fig. 1).

### Model Performance

**Fig. 2:**
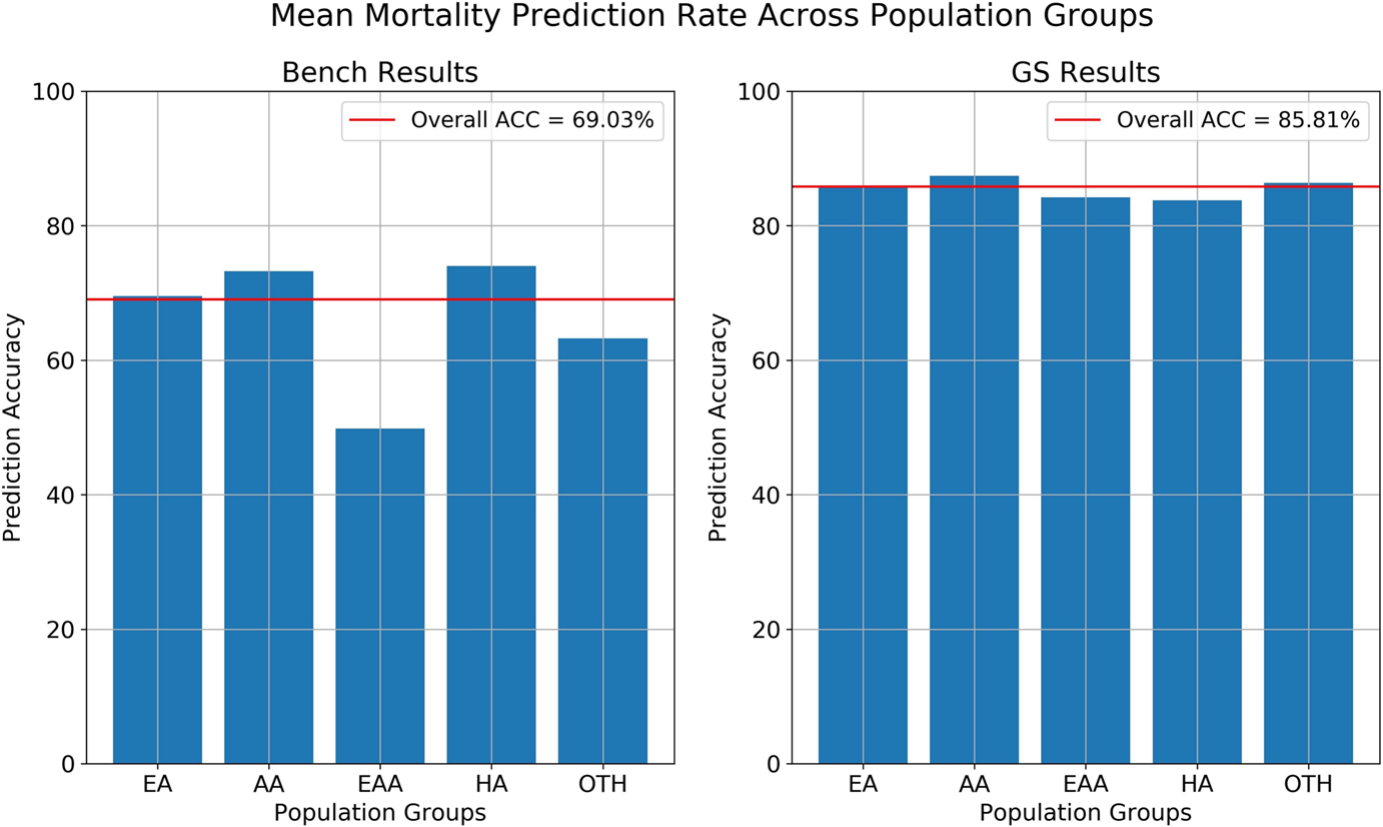
Mean prediction comparison of Benchmark and GS models across different racio-ethnic populations. Bars indicate the model accuracy per population groups, while the dashed lines show the overall model accuracies. Unlike Benchmark, the GS model exhibits more uniform performance, favoring each population group approximately equal as shown in previous ROC curves (Fig. 1)

Two significant outcomes are observed when comparing the post-GS-trained model accuracy with the benchmark model. The model’s accuracy demonstrates greater parity across racio-ethnic groups indicating bias reduction as shown in Fig. 2. Furthermore, we have observed a significant increase in prediction accuracy for the post-GS-trained model overall.

For a more thorough examination, the Receiver Operating Characteristics (ROC) curves and respective Area Under Curve (AUC) scores for each ethnicity in both benchmark and GS settings are measured, and results are illustrated in Fig. 1. The AUC score provides a more comprehensive measure of a model’s performance and ability to distinguish between positive and negative examples in a binary classification task by computing the trade-off between the true positive rate (sensitivity) and the true negative rate (specificity). We observe every ethnicity’s AUC score not only improves but additionally converges to a more uniform value when GS is applied.

### SHAP

**Fig. 3:**
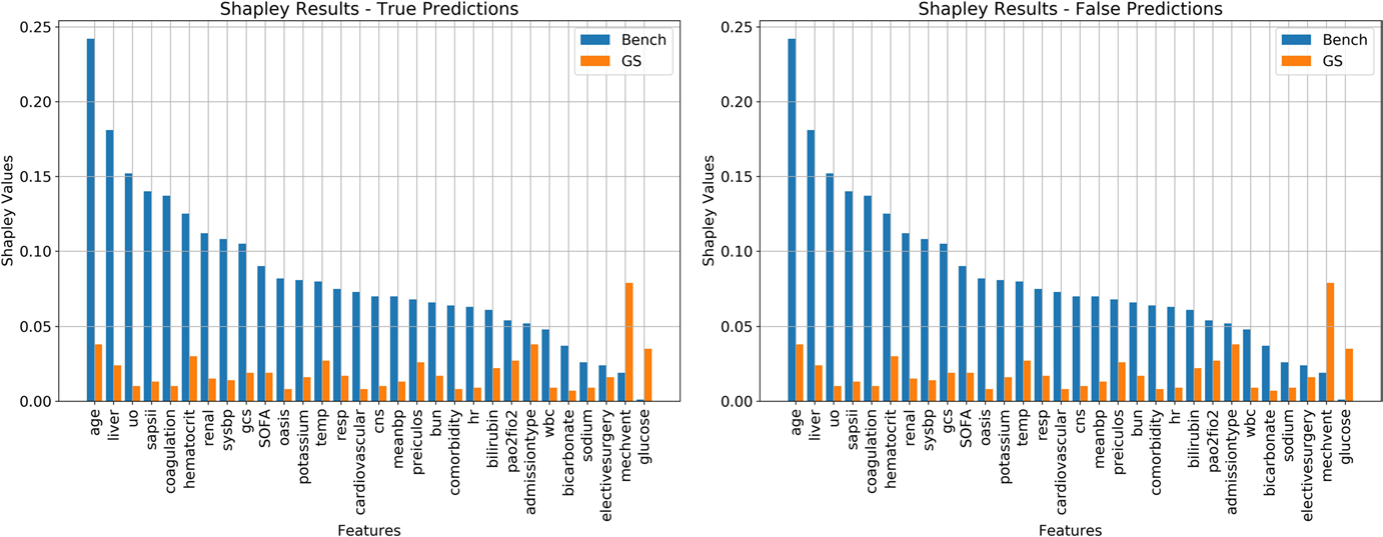
Comparison of Feature Importance between Benchmark and GS models. The bar graphs depict the distribution of feature importance for each model. For both True and False predictions of the models, the features preprocessed with the GS transformations have more balanced importance scores indicating a more equitable consideration of features during the model training and prediction phases

Figure 3 provides a compelling visual representation of the model’s feature contributions both before and after the application of the Gerchberg-Saxton (GS) transformations. The distinctions between the GS-trained model and the benchmark model are evident, with the GS-trained model exhibiting a notably more balanced distribution of feature importance.

While the plot on the left showcases feature contribution for True (1) predictions, the right side highlights the contribution for False (0) predictions. In each case, the blue bars map the feature contribution from the benchmark model, whereas the orange bars reflect the contributions post-GS transformation.

A pivotal observation from this analysis is the reshuffling of feature significance in the GS-trained model, pointing to the model’s enhanced capability to generalize across diverse racio-ethnic groups. This outcome aligns with the primary intent behind GS transformations: to attain a harmonious distribution of information among features. This not only leads to a leveling of the playing field, where features pertinent to minority populations gain prominence, but it also results in predictions that are less skewed by the dominant influence of major populations.

Further deep diving into specific features, it is intriguing to note the amplified contributions of “mechanical ventilation” and “glucose scores” in the post-GS model. While these features were either marginal or completely absent in their influence on the benchmark model, post-transformation, they emerged as significant predictors. This shift underscores the potency of the GS transformations in spotlighting critical features that might have been overshadowed in the initial data representation. Hence, the overarching implication is that GS transformations not only bolster the fairness of the model but also enhance its predictive robustness by ensuring that no crucial feature is side-lined.

### Information Entropy

**Fig. 4:**
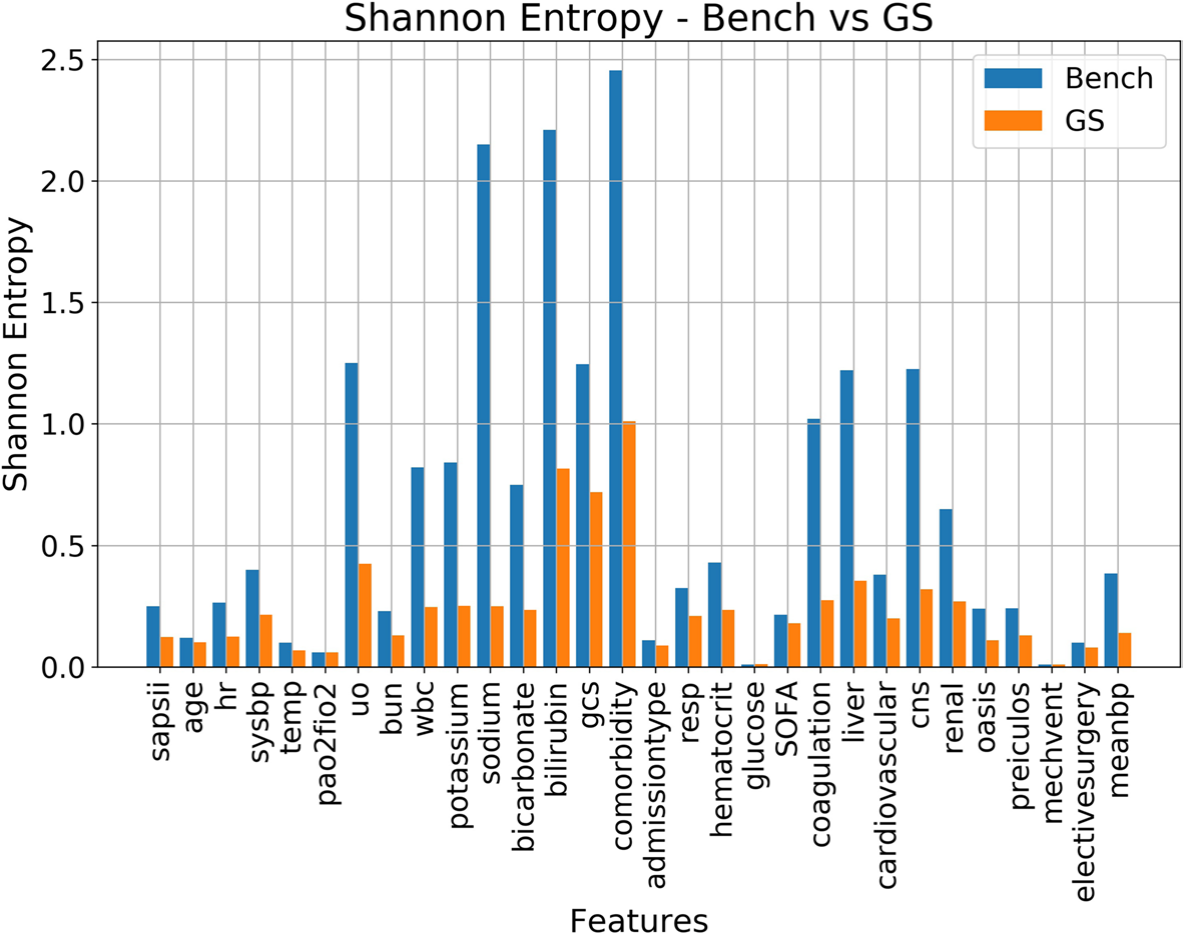
Information Entropy comparison between original and GS transformed feature values. Despite some loss, the information contained per features are more uniformized via GS transformations. This, along with the feature importance results (Fig. 3), suggests that the desired information distribution effects have been achieved successfully via GS transformations

Figure 4 provides a comparative analysis of Shannon entropy across the features, both prior and subsequent to the Gerchberg-Saxton (GS) transformations. Shannon entropy, fundamentally, measures the uncertainty or randomness of a dataset. The displayed differences between the pre- and post-GS models in this figure underscore the significant reduction in this uncertainty post of the GS application.

The height of the bars in the graph provides insight into the information entropy levels of the respective features. It is evident from the graph that the range of Shannon entropy values for the benchmark dataset spanned from 0 to approximately 2.5, reflecting a more variable degree of uncertainty. Conversely, post-GS transformation, this range becomes notably narrower, with values primarily clustering within the 0 to 1 bracket. This narrowed range attests to the diminished uncertainty and a consequent enhancement in information consistency across the features.

An inference to be drawn here is the positive influence of GS transformations on the MIMIC-III vital signs dataset. Through these transformations, the model’s training exhibits heightened consistency. This translates into a greater reliability in the resulting predictions, an aspect that holds paramount importance, especially when these predictions pertain to mortality rates. Such predictions are intertwined with critical healthcare decisions, and any stride toward their accuracy is of intrinsic value.

In sum, the portrayal of Shannon entropy in Fig. 4 underscores the transformative potential of GS. By curbing the inherent uncertainty and amplifying the consistency in the features during model training, GS transformations present a promising avenue for bias mitigation. This not only engenders a higher fidelity in predictions but also substantiates the overarching endeavor of utilizing MIMIC-III vital signs data for mortality rate predictions with utmost precision and reliability.

### Fairness Constraints

Both the Demographic Parity and Error Rate Parity comparisons shed light on the improvements in fairness achieved by the GS-influenced model when juxtaposed with the benchmark model.

**Fig. 5:**
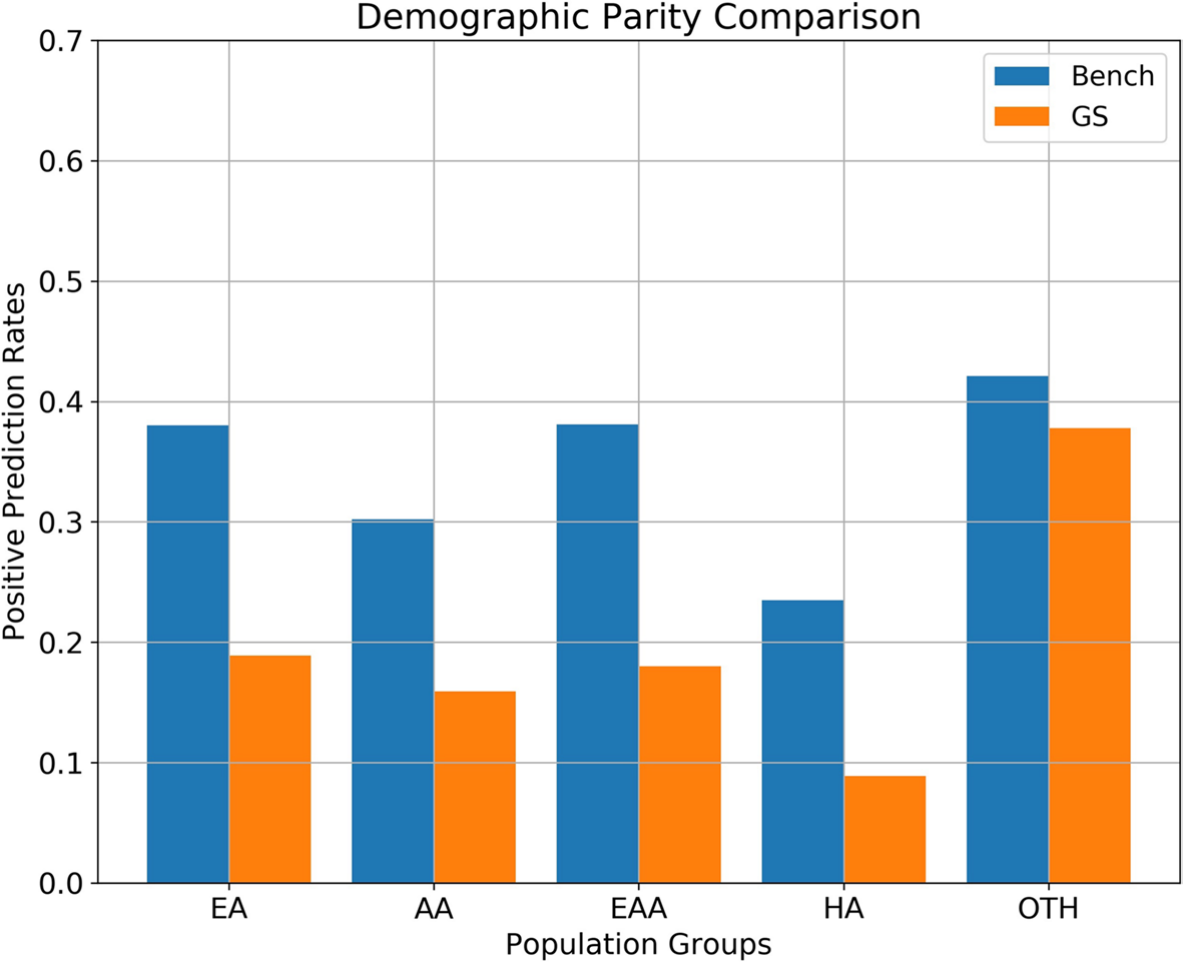
The GS model exhibits less variability in positive prediction rates across different racio-ethnic groups, indicating its predictions are less discriminatory against any demographic groups. This suggests enhanced fairness in model predictions across various demographic groups

In Fig. 5, Demographic Parity comparisons illustrate that the GS-influenced model yielded a more evenly distributed positive prediction rate across various racio-ethnic groups. The benchmark model, on the other hand, demonstrated disparities in its positive predictions, particularly among minority groups. The GS model’s balanced prediction rate across groups underscores its potential to counteract biases and produce more equitable prediction outcomes among sensitive groups [26].

**Fig. 6:**
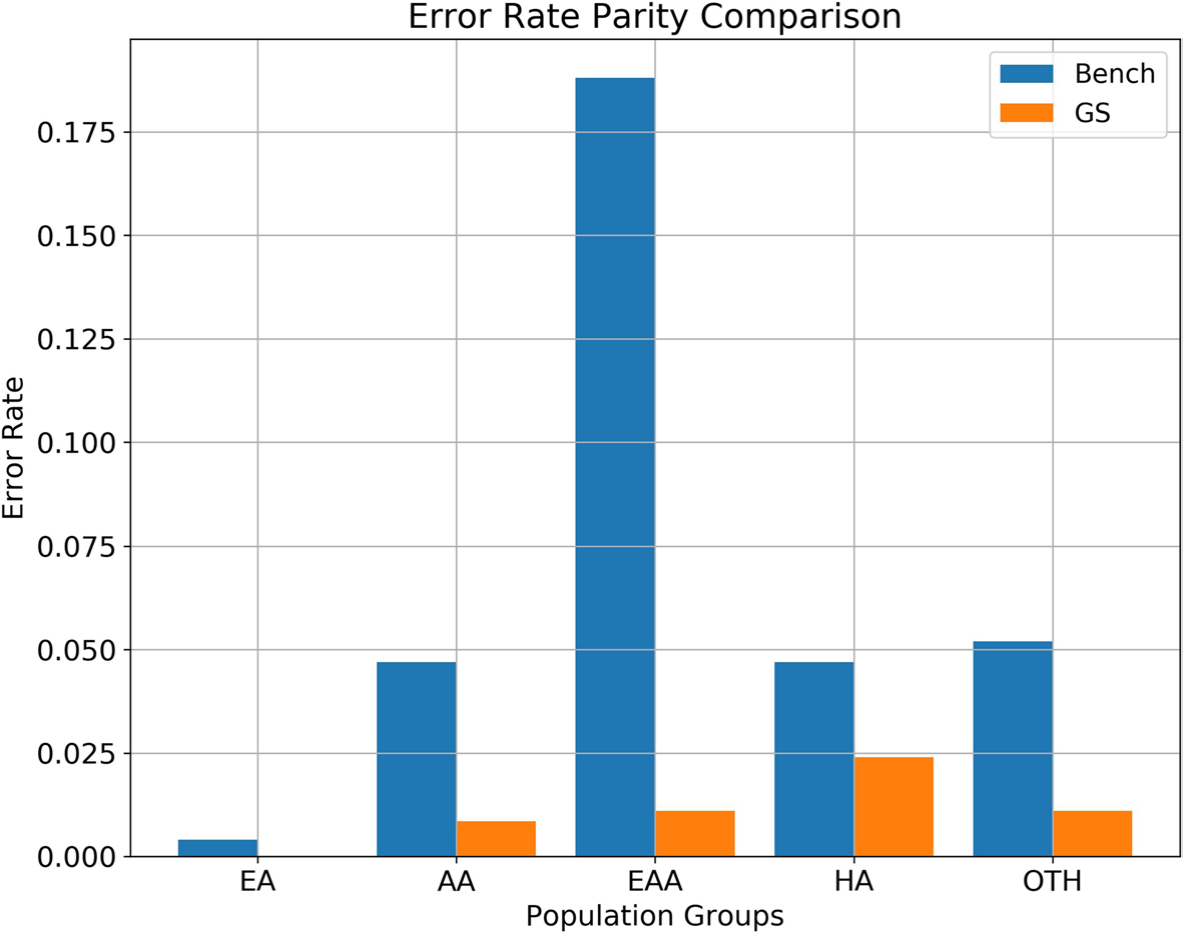
The chart illustrates the two models’ error rates across various racio-ethnic groups. Lower and more consistent error rates in the GS-influenced model suggest improved fairness and reduced disparity in prediction accuracy across different demographic groups

Shifting to Fig. 6, the Error Rate Parity comparison corroborates the GS model’s efficacy. A salient observation is the error rate within the European American (EA) group. While the benchmark model, possibly influenced by the predominance of EA in the dataset, already showcased a reduced error rate, the GS-influenced model achieved even greater accuracy for this group. The near-total eradication of error in the EA group is remarkable, signifying the model’s robustness, even though achieving such precision across all groups in every dataset might not always be possible. For the other racio-ethnic categories, namely AA (African American), EAA (East Asian American), HA (Hispanic American), and OTH (Other), the GS-influenced model consistently outperformed the benchmark by reducing error rates. This consistency underpins the GS model’s proficiency in providing balanced, fair, and unbiased predictions across a spectrum of groups.

In summation, the incorporation of the GS algorithm into the MIMIC-III dataset has undeniably elevated the fairness quotient of the model’s predictions among the highlighted race-ethnic groups. Subsequent studies can probe deeper into the mechanism of the GS transformation and elucidate the precise modifications that facilitated these advancements in fairness.

## Discussion

In our exploration, we employed extant tools innovatively to spotlight bias mitigation across diverse racio-ethnic populations in a DL model. This model was honed on features exhibiting high correlations, leveraging sophisticated frequency domain operations. Using the MIMIC-III database, known for its inherent selection biases and disparities, we applied the Gerchberg-Saxton (GS) algorithm to pioneer the application of information distribution on data.

At its core, the GS algorithm is a model-agnostic technique, centering on data transformation rather than tweaking the model. This accentuates the fact that model alterations would not innately boost the bias-deterrence prowess of the GS algorithm. Given that the sampling bias discussed stems from the dataset rather than a specific model type or structure, the GS’s role becomes pivotal. Serving as a potent data transformation instrument in our research, the GS algorithm, when applied, acts as the forerunner of bias mitigation at the dataset level.

However, it is prudent to highlight some intrinsic limitations to our approach. Tackling extremely voluminous datasets poses a computational challenge. Subjecting such a dataset to the GS transformation in its entirety is often unfeasible. Consequently, data must be segmented and processed in batches. Herein lies a pivotal consideration: information distribution within individual batches by GS does not benefit subsequent batches. Therefore, curating these batches demands meticulous attention, ensuring a near-equitable representation of all racio-ethnic groups within each batch. Although this may not guarantee a flawless, uniform information dissemination across the complete dataset, conscientiously distributing similar racio-ethnic samples across batches can approximate it closely.

A second limitation surfaces in the interpretability of GS-transformed data. The transformation outputs a structurally altered version when compared to the original. Particularly in biological applications, this metamorphosed data might be alien to conventional representations. Nevertheless, as elucidated in our results and illustrated graphically, these structural deviations do not impede AI models. In certain instances, the resultant less sparse data can even enhance the model’s efficacy. Additionally, the original dataset remains accessible for the purpose of biological interpretation of the results. It is important to note that the GS algorithm merely disseminates the information without inducing any alterations to the content represented in the dataset.

To achieve a more even-handed and dependable model prediction rate, the GS algorithm’s strategy aimed to disperse information from features that predominantly informed model predictions for the European-American groups to all other racio-ethnic categories. Employing the GS algorithm pre-data partitioning into training, testing, and validation subsets, ensured an egalitarian information dissemination across all subsequent data subsets.

Post-GS model analysis unveiled two paramount revelations vis-à-vis the benchmark model. Firstly, the GS algorithm’s prowess in equalizing feature magnitudes in the frequency domain acted as a bias counteragent, leveling the prediction accuracy and AUC scores playground across all racio-ethnicities. Secondly, the post-GS model showcased heightened prediction accuracy and AUC scores for all ethnic factions.

Our comparative study between benchmark and GS-influenced models, especially in the domains of Demographic and Error Rate Parity, sought to vouchsafe model fairness across sensitive demography. Figures 5 and 6 corroborate the GS model’s superiority, highlighting its reduced biases and enhanced fairness.

In summation, while our study lays the foundation in ethically mitigating biases in medical DL model applications, it is paramount to further unearth the GS algorithm’s full potential in bias deterrence and its adaptability across diverse medical data facets, beyond just vital signs.

## Conclusion

This study demonstrates a method for bias mitigation utilizing advanced frequency domain operations via the Gerchberg-Saxton algorithm on biomedical data. With the application of the GS algorithm on the MIMIC-III, we illustrated the effects of the information distribution on mortality rate prediction accuracy, which results in a more uniform and equitable (and in some cases increased) model prediction for various racio-ethnic groups. We performed SHAP calculations and utilized Shannon entropy for a deeper analysis of GS’s effect for validation. The results of these analyses verify that a more uniform feature contribution indicates a more equitable training process. While further research is required to investigate the full capacity and performance of the GS algorithm in other settings and with other modalities to explore its full potential in medical applications, we believe that the implications of our study are significant and have the potential to advance current ongoing efforts investigating bias mitigation.

## Figures and Tables

**Fig. 7:**
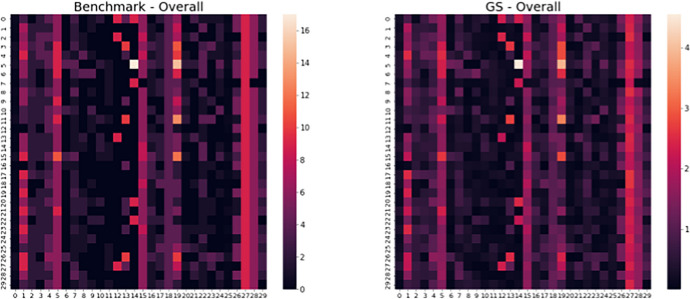
Figure above illustrates the overall heatmap comparison of benchmark and GS-transformed training datasets to validate the preservation of local maxima and minima locations on post-GS datasets

**Fig. 8:**
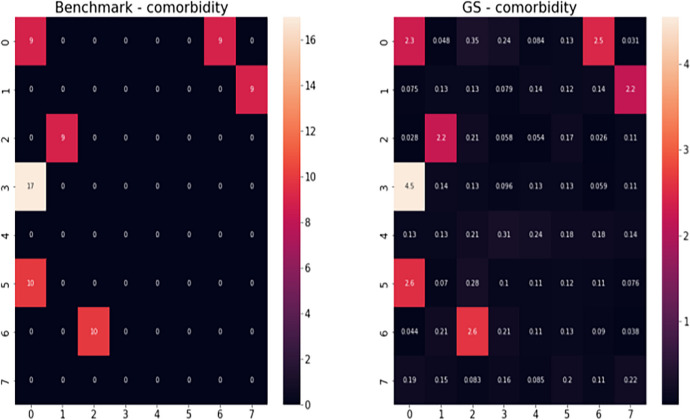
Figure above illustrates the feature-by-feature heatmap comparison of benchmark and GS-transformed training datasets to validate the preservation of local maxima and minima locations on post-GS datasets

## Data Availability

Publicly available MIMIC-III database is utilized in this study. The MIMIC-III database (version 1.4) can be found at https://mimic.physionet.org/

## Appendix

### Data Comparison via Heatmap

In this section, we will be illustrating our results in terms of data usability after Gerchberg-Saxton transformations. As mentioned earlier, in order to determine the dataset usability after GS transformations, we have compared the input (benchmark) and output (post-GS) matrices of the Gerchberg-Saxton algorithm first overall side by side, and then individual feature by feature using heatmaps. We have finalized the usability of the output matrix based on the preservation of the local minima and maxima locations before and after the GS algorithm to assess the transformed dataset’s ability to preserve essential features for predicting mortality rates. However, the obtained results hold true and consistent for all other feature-by-feature comparisons.

Appendix Fig. 7 is the overall comparison for dataset before and after the Gerchberg-Saxton transformations. For better visual quality, we have selected the first 50 patients from both benchmark and GS-transformed datasets. As illustrated in Appendix Fig. 7, in the heatmap comparison of these two datasets, the local maxima and minima locations are preserved for all elements confirming the usability of the GS-transformed dataset on model training for mortality rate prediction.

Similarly, Appendix Fig. 8 illustrates the feature-by-feature comparison of the dataset before and after GS transformations. We have randomly selected a feature among all 30 trainable features to illustrate the results. As stated above, in all other feature comparisons, the local maxima and minima locations are preserved, which is an additional validation criterion for the usability of the post-GS datasets on model training.
